# Human pluripotent stem cell-derived hepatic progenitors exhibit a partially hypoimmunogenic phenotype and actively inhibit immune responses

**DOI:** 10.3389/fimmu.2025.1507317

**Published:** 2025-02-25

**Authors:** Malika Gantier, Séverine Ménoret, Angélique Fourrier, Frédéric Delbos, Tuan Huy Nguyen, Ignacio Anegon

**Affiliations:** ^1^ GoLiver Therapeutics, Nantes, France; ^2^ Nantes Université, Inserm, CNRS, SFR Santé, Inserm UMS 016 CNRS UMS 3556, Nantes, France; ^3^ Nantes Université, INSERM, Center for Research in Transplantation and Translational Immunology, UMR 1064, Nantes, France

**Keywords:** pluripotent stem cells, tolerance, transplantation, liver, hepatic progenitors

## Abstract

**Introduction:**

GStemHep cells are human cryopreserved hepatic progenitors derived from pluripotent of stem cells (GStem cells) using a cGMP-compliant protocol. They were highly effective in rescuing mice from acute liver failure.

**Methods:**

The objective of this study was to analyze the immunogenicity and immunoregulatory properties of GStemHep cells.

**Results:**

As compared to GStem cells, GStemHep cells showed complete loss of HLA-I (ABC) and they lacked of expression of HLA-II, HLA-G, HLA-E and PD-L1. GStemHep cells also showed increased expression of CD47, maintained high expression of indoleamine 2,3-dioxygenase (IDO) and heme oxygenase-1 (HO-1) and reduced expression of CD200. In comparison with GStem cells, GStemHep cultured in inflammatory conditions increased the expression of PD-L1, CD200, HO-1, HLA-E, CD47 and HLA-I (ABC) as well as maintained expression of IDO and were negative for HLA-II and HLA-G. GStemHep culture in basal or inflammatory conditions has a low or absent immunogenic activity on T cells, associated to a suppressive effect on proliferation partially mediated by IDO. We observed phagocytosis of GStemHep by macrophages that was partially inhibited by CD47 expression. NK cells were activated by resting GStemHep cells. Upon culture in inflammatory conditions that induced expression of HLA-I molecules in GStemHep cells NK cell activation was reduced. Thus, GStemHep cells are partially hypoimmune cells due to the expression of several immune checkpoint inhibitors and the absence of HLA-I molecules. In inflammatory conditions, the expression of several of these molecules was increased but also of HLA-I that could be immunogenic for T cells but it was inhibitory for NK cells.

**Discussion:**

GStemHep cells show a favorable immunological profile for their use as allogeneic off-the shelf treatment of liver diseases with loss of hepatocyte function.

## Introduction

Cell transplantation is becoming an increasingly important alternative to organ transplantation to overcome the shortage of donors. In the field of liver pathology, primary hepatocyte transplantation has already demonstrated its biosafety and medium-term success by improving patients’ liver functions in acute and chronic liver disorders (1–3). However, current culture conditions fail to efficiently expand human primary hepatocytes *in vitro* to a sufficient scale for the treatment of many patients, resulting in a continual need for multiple liver donors. We therefore need another source of cells, of consistent quality, available at all times and on a large scale.

These other cell sources under development for liver cell transplantation include pluripotent stem cells (PSCs) (4). Because of their capacity for self-renewal and differentiation into any cell type, PSCs such as embryonic stem cells (ESCs) and induced pluripotent stem cells (iPSCs) hold great promise in regenerative medicine. PSC-derived hepatocytes showed potential in the treatment of several liver diseases. Indeed, we and other groups have reported the therapeutic efficacy of these cells into Crigler-Najjar (5, 6) and acute liver failure (ALF) models (7–10). Previously, we described the production, cryopreservation and characterization of human PSC-derived hepatic progenitors called GStemHep, also showing potent therapeutic effect *in vivo* in two different ALF models (7). In this previous study, we addressed several issues related to cell therapy drugs, such as large-scale production under GMP conditions and cryopreservation of cells to be available at all times (7).

The use of patient-derived autologous iPSCs is theoretically an interesting approach to overcome immune rejection. However, the generation of autologous iPSCs is a costly, complex and time-consuming process that preclude its use in acute clinical situations (11). This is why the allogeneic approach with off-the shelf available cells is gaining favor. One of the main challenges of this strategy of cell therapy is the inherent risk of immune rejection of the allogeneic cells by the recipient (12). In the case of GStemHep, we have demonstrated that the very rapid mode of action of the cells was sufficient for a therapeutic effect in acute liver failure even before acute rejection could occur (7). However, in the case of chronic diseases of the liver, more than one cell treatment may be required and an allogeneic immune response against the therapeutic cell product may destroy the cells too rapidly impeding cell implantation into the liver likely necessary for a successful outcome. Several strategies are being implemented or explored to limit immunogenicity and allogeneic cell rejection of ESCs or iPSCs, such as banking ESCs or iPSCs with HLA covering the majority of the population, induction of tolerance in the recipient by targeted manipulation of specific costimulatory pathways, genome editing of transplanted cells to reduce the expression of immunogenic molecules, or on the contrary, increase that of immunoregulatory molecules (13–15).

The needs and approaches used will depend closely on the immunomodulatory properties of the transplanted cells. Indeed, the source of origin of the cell product has a potentially significant impact on rejection. For example, ESCs and iPSCs require different methodologies to generate the final differentiated cell product, and iPSC have been shown to be more immunogenic (16). Unlike ESC-derived differentiated cells, the reprogramming procedure for iPSC generation could result in aberrant antigen presentation due to the retained partial epigenetic memory of the original somatic cells (17). Additionally, differentiation from iPSCs into different cell types may also determine differences in the expression of potentially antigenic or immunomodulatory molecules that can reproduce immunogenic properties of the endogenous cell counterpart (18). Therefore, it is imperative to investigate the immunogenicity of a precise cell type derived from a given PSC allowing to at least partially predict immune rejection prior to transplantation.

Here, we analyzed the expression by GStemHep of different molecules potentially involved in allogenicity and immunoregulation (15), such as conventional human leukocyte antigens class I (HLA-I ABC) and class II (HLA-DR) major histocompatibility complex (HLA) molecules, as well as non-conventional class I HLA molecules (HLA-E/G). We also analyzed the expression of several important immunoregulatory molecules, such as CD47 (19–21) and CD200 (22–24) that inhibit macrophage and NK cell function as well as PD-L1 (25), IDO (26–28) and heme oxygenase-1 (HO-1) (29–31) that inhibit DC, T and NK cell functions. The phenotype of GStemHep after differentiation from GStem cells resulted in an almost complete silencing of HLA-I (ABC) expression and lack of expression of unconventional HLA class I (HLA-E/G), as well as HLA-II molecules. GStemHep continued to express IDO and HO-1 at high levels, increased their expression of CD47 and maintained CD200 expression, albeit at reduced levels compared to GStem cells. We observed a low or absent immunogenic activity of GStemHep cells on T cells, associated to a suppressive effect on proliferation partially mediated by IDO. We observed phagocytosis of GStemHep by macrophages that was partially inhibited by CD47 expression by GStemHep cells. NK cells were activated by resting GStemHep cells and upon culture in inflammatory conditions that induced expression of HLA-I molecules NK cell activation was reduced.

Overall, GStemHep cells have low immunogenicity and active immunoregulatory mechanisms that suggest a favorable immunological profile for their use as an allogeneic off-the shelf cell therapy of acute and chronic hepatic diseases.

## Methods

### GStemHep cells

GStemHep were produced from a cGMP human PSC line, as previously described (7) and stored in a cryopreservation solution (Cryostor10 - ThermoFisher) at -150°C. For all experiments, GStemHep were thawed in a water bath at 37°C for 1 min, transferred to RPMI-1640 medium supplemented with B27 (Life Technologies) and centrifuged (200g - 10min) for washing before use. When using thawed GStemHep, the cell pellet was directly transferred to the appropriate medium for the experiment. For fresh GStemHep, cells were seeded in culture plates coated with iMatrix-511 (Amsbio) in RPMI-1640 supplemented with B27, 10% fetal bovine serum (FBS - Eurobio), 10 μM Rock inhibitor Y-27632 (StemCell Technologies), 3 μM CHIR-99021 (Stem Cell Technologies) and 20 ng/mL HGF (Miltenyi Biotec). Cells were cultured 1 to 3 days before use. For inflammatory GStemHep, 24 hours after seeding, medium was changed with RPMI-1640 supplemented with 1% Glutamine, 5% human AB serum, 100U/mL TNFα, 10ng/mL IL1β and 20ng/mL IFNγ (Miltenyi) for 24 hours. For CD47 overexpression, 24 hours after seeding, a lentiviral vector carrying the CD47 transgene overexpression cassette was added for 48 hours.

### PBMCs isolation

The peripheral blood mononuclear cells (PBMCs) used in the various experiments were derived from blood supplied by the French Blood Establishment (EFS-Nantes). PMBCs were isolated by density gradient centrifugation (2000RPM - 20min) on Ficoll (Lymphocyte Separation Medium^®^ - Eurobio). After recovery of the cell ring and washing in PBS, the cells were centrifuged (1000RPM - 10min) to remove platelets. The harvested PBMCs pellet is then washed twice by centrifugation in PBS (1400RPM - 7 min) before use.

### Coculture assays to evaluate lymphocytes activation and proliferation

GStemHep were seeded at different densities 24h before coculture. Fresh blood PBMCs were labeled with the proliferation marker CPD670 (eBioscience™ Cell Proliferation Dye eFluor™ 670 - ThermoFisher) according to the supplier’s instructions. Labeled PBMCs were then added to plated GStemHep at different PBMC: GStemHep ratios (1:1 and 1:5) in co-culture medium consisting in RPMI-1640, 1% Glutamine and 5% human AB serum with or without 100U/mL TNFα, 10ng/mL IL1β and 20ng/mL IFNγ (Miltenyi). In parallel with coculture, PMBCs were cultured alone for the negative control or in the presence of 25ng/mL PMA (phorbol myristate acetate) and 1μg/mL ionomycin or CD3/CD28 activation beads (1 bead per 40 PBMC_ CD3/CD28 Dynabeads™ - ThermoFisher Scientific) for the positive stimulation control. For IDO inhibition experiments, coculture was performed in RPMI-1640/Glutamine/human AB serum medium with 200μM of the inhibitor 1-methyltryptophan (Sigma) and CD3/CD28 activation beads (1 bead per 40 PBMC). After three days of coculture, CD3+ T cell proliferation (CPD670) and activation (CD69/CD25/HLA-DR) were analyzed by flow cytometry.

### Coculture assays to evaluate macrophages phagocytosis

Monocytes were sorted from the PBMC population using the Classical Monocyte Isolation Kit and the autoMACS^®^ Pro Separator (Miltenyi). Monocytes were then cultured for 5-6 days in RPMI-1640 medium with 10% FBS and 100 ng/mL macrophage colony-stimulating factor to differentiate them into macrophages. GStemHep were seeded for 72 hours, then dissociated with TrypLE™ (ThermoFisher) or used directly after thawing. *Cytometry analysis:* M0 macrophages and GStemHep were labeled with the proliferation markers CPD670 and CPD450 respectively (eBioscience™ Cell Proliferation Dye eFluor™ 670/450 - ThermoFisher) according to the supplier’s instructions. Labeled cells were then cocultured in ULA (Ultra-low Attachment) 96-well plates in RPMI-1640 medium with 1% Glutamine and 5% human AB serum at 1:1 ratio. Phagocytosis was evaluated as described (32) after two hours of coculture, cells were labeled with a cell viability marker and analyzed by flow cytometry. For CD47 overexpression experiments, GStemHep were seeded for 24 hours, then transduced with the lentiviral vector for 48 hours prior to coculture. For CD47 inhibition experiments, labeled GStemHep were treated for 1 hour with the blocking anti-CD47 antibody or its isotype control in ULA 96-well plates plate before adding macrophages for coculture. *Microscopy analysis:* M0 macrophages and GStemHep were labeled with the CellTracker™ Green and red pHrodo™ SE respectively (ThermoFisher) according to the supplier’s instructions. Labeled cells were then cocultured in ULA plates in RPMI-1640 medium with 1% Glutamine and 5% human AB serum at 1:1 ratio. After two hours of coculture, cells were observed under a Zeiss fluorescence microscope.

### Coculture assays to evaluate NK cell activation and degranulation

NK cells were sorted from the PBMC population using the NK Cell Isolation Kit and the autoMACS^®^ Pro Separator (Miltenyi). NK cells were then cultured for 24 hours in RPMI-1064 medium with 10% FCS and 500 IU/mL human IL2. GStemHep cells were used directly after thawing or pretreated in inflammatory medium. NK cells and GStemHep were cocultured in the presence of CD107a-FITC antibody (BD Biosciences) in RPMI-1640, 1% Glutamine and 10% SVF at NK: GStemHep ratios of 1:1 and 1:2. After five hours of coculture, cells were labeled with a cell viability marker and expression of membrane CD107a on NK cells (CD56+) was analyzed by flow cytometry.

### Immune cells characterization by flow cytometry

The phenotype of the various immune cells used in coculture assays (see above) was analyzed by flow cytometry. Cells were incubated with a viability marker (LIVE/DEAD Fixable Blue, Aqua or Yellow stain - ThermoFisher) for 20 min at 4°C. A Fc receptor saturation step was performed by incubation in FcBlock (ThermoFisher) for 20 min at room temperature. The cells were then incubated with the different antibodies (**Supplementary Table S1**) diluted in PSE for 30 min at 4°C. Flow cytometry analysis was performed with a FACSCanto II or CELESTA cytometer (BD Biosciences) and Flow Jo analysis software (Tree star).

### GStemHep characterization by flow cytometry

Cells (immediately after thawing or thawed and cultured cells) were incubated with a viability marker (LIVE/DEAD Fixable Blue stain, ThermoFisher) for 20 min at 4°C. A saturation step, in PSE (PBS/2% FBS/2mM EDTA) for membrane labeling or in permeabilization buffer (PBS/0.1% BSA/0.1% Saponin) for cytoplasmic labeling, was performed for 20 min at 4°C. The cells were then incubated with the antibody (**Supplementary Table S1**) diluted in saturation buffer for 30 min at 4°C, and finally resuspended in PSE. In the case of transduced cells, a fixation step was performed before flow cytometry by incubation in paraformaldehyde for 15min at 4°C. Flow cytometry analysis was performed with a FACSCanto II (BD Biosciences) and Flow Jo software (Tree star).

### Statistical analysis

Statistical analysis was achieved with GraphPad Prism 5 software. All results are expressed as the mean ± SEM. Mann−Whitney, Wilcoxon and two-way ANOVAs were used when appropriate and are indicated in each figure.

## Results

### GStemHep have a weakly immunogenic profile

**Figure 1:**
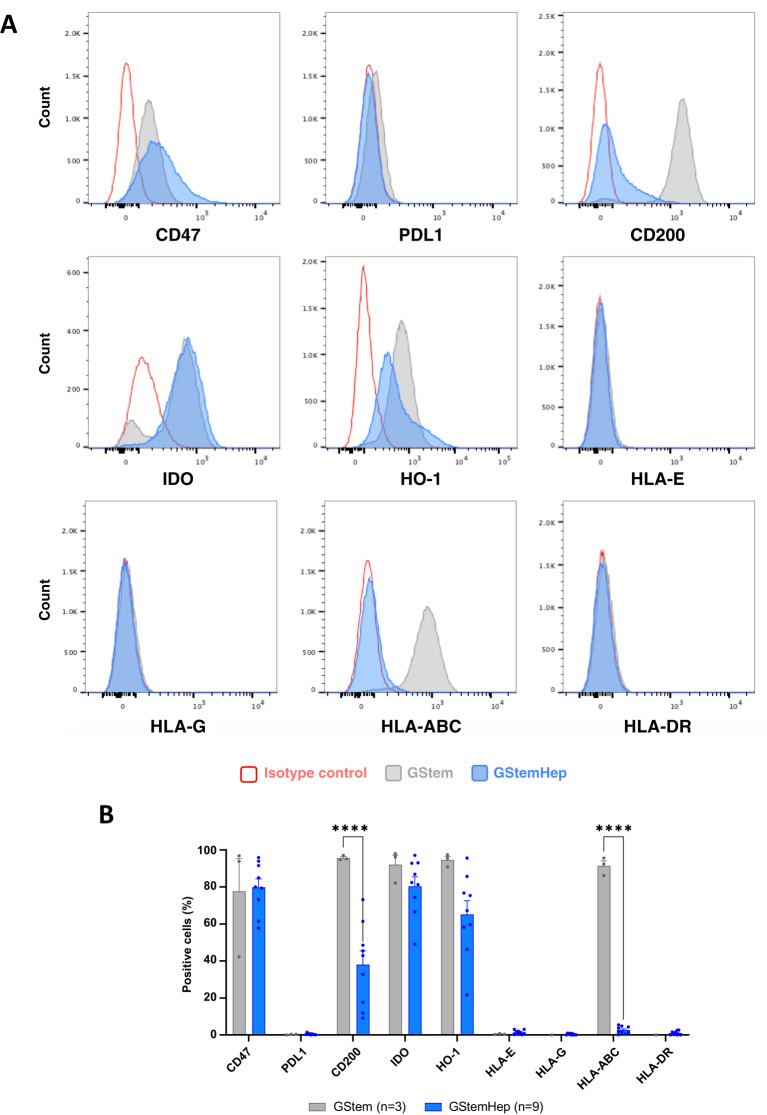
Expression profile of immune markers in GStemHep and GStem cells. The expression of various molecules (CD47, PDL1, CD200, IDO, HO-1 and HLA- E/G/ABC/DR) involved in the cellular immune response was analyzed in thawed cells by flow cytometry. **(A)** Representative FACS histogram of the pluripotent stem cell line GStem (grey) and representative GStemHep cells from one production batch (blue). Cells incubated with isotype control are in red. **(B)** Percentage expression of different markers in several productions of GStemHep (n=17) after thawing compared with the pluripotent stem cell line GStem (n=3, mean ± SEM, two-way ANOVA test, ****p < 0.0001).

To predict the immunogenicity of GStemHep *in vivo*, the immunophenotypic profile of these cells was characterized by flow cytometry. The expression of various molecules involved in the activation or regulation of the immune system was assessed on different GStemHep production batches immediately after thawing, as they would be used in the clinic, and on the PSC line before differentiation (called GStem) (**Figures 1A, B**). Histograms of the cytometric analysis for the GStem line and for a representative GStemHep production are shown in **Figure 1A**. The average expression of these different markers in the GStemHep production batches (n=9) is shown and compared with the GStem line (n=3) in **Figure 1B**. HLA-I molecules were expressed by 91.4 ± 2.8% of the cells of the GStem line used for differentiation into GStemHep and this expression was greatly reduced in GStemHep and averaged 2.6 ± 0.7%. Differentiation of GStem into GStemHep also resulted in a significant decrease in CD200 expression (95.6 ± 0.7% vs. 38.1 ± 7.4%). Neither GStemHep nor GStem expressed unconventional HLA class I (HLA-E/G) or class II (HLA-DR) molecules (detection threshold < 1%). Differentiation did not significantly affect basal CD47 (77.6 ± 17.7% vs. 79.8 ± 4.5%), IDO (92.1 ± 5.0% vs. 80.4 ± 5.1%) or HO-1 (94.6 ± 2.0% vs. 65.2 ± 7.4%) expression found in GStem. Finally, neither GStem, nor GStemHep cells expressed PD-L1.

Analysis of the intensity of expression for these molecules showed that differentiation of GStem into GStemHep significantly decreased CD200 expression and did not modify CD47, IDO and HO-1 levels of expression (**Supplementary Figure S1A**).

In conclusion, GStemHep cells present a phenotype with low immunogenicity, characterized by an absence of expression of HLA-I and -II molecules and expression of immunoregulatory molecules such as CD200, CD47, IDO and HO-1.

### GStemHep phenotype in inflammatory conditions

**Figure 2:**
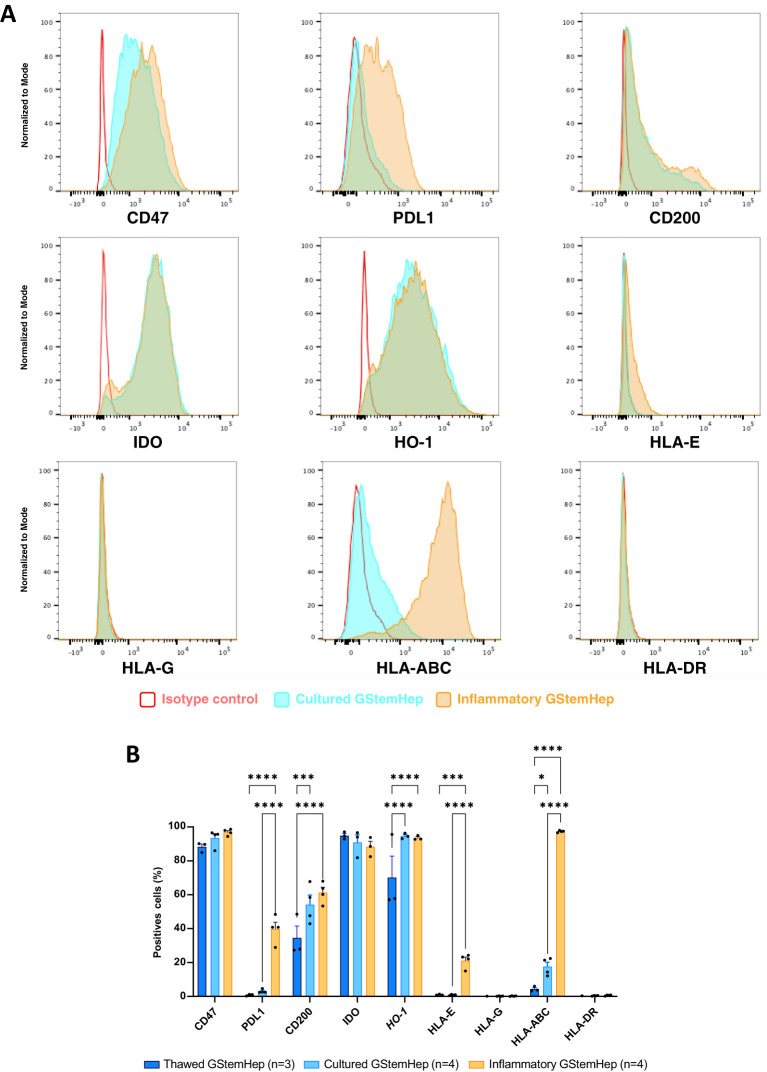
Expression profile of immune markers in GStemHep after inflammatory treatment. GStemHep were thawed and plated in culture medium with (inflammatory GStemHep) or without (cultured GStemHep) inflammatory cytokines (TNFα/IL1β/IFNγ). The expression of various molecules (CD47, PDL1, CD200, IDO, HO-1 and HLA-E/G/ABC/DR) involved in the cellular immune response was analyzed by flow cytometry in both culture conditions and compared with thawed cells. **(A)** Representative FACS histogram of thawed (dark blue), cultured (light blue) and inflammatory (orange) GStemHep. Cells incubated with isotype control are in red. **(B)** Percentage expression of different markers in several productions of GStemHep (n=4) after culture compared with thawed cells (n=3) and cultured cells in basal conditions (n=4). (two-way ANOVA test, *p < 0.05; ***p < 0.001; ****p < 0.0001).

Most liver diseases, and acute liver failure in particular, are characterized by major inflammation in the liver, so we assessed the phenotype of GStemHep cultured in inflammatory conditions since this is the environment in which the cells will be placed upon transplantation into the liver. Thawed GStemHep, the ones that will be injected in patients, were cultured for 24 hours in medium with or without inflammatory cytokines (TNFα/IL1β/IFNγ), to mimic the inflammatory conditions *in vivo*. Then, the expression of the molecules previously analyzed was assessed on both culture conditions and thawed cells without culture (**Figure 2**). Representative histograms of the cytometric analysis of GStemHep cells cultured in basal and inflammatory conditions are shown in **Figure 2A**. The average expression of different markers in these culture experiments is shown and compared with the cells thawed and analyzed immediately in **Figure 2B**. As compared to thawed cells, cultured cells showed a low but significant increase in the expression of CD200 (34.6 ± 7.0% vs. 54.2 ± 5.6%), HO-1 (70.1 ± 12.8% vs. 94.6 ± 0.9%) and HLA-I (ABC) (4.4 ± 0.9% vs. 17.8 ± 2.6%). The rest of the markers of thawed vs. cultured cells were unchanged. In inflammatory conditions and compared to thawed cells significantly expressed higher levels of PD-L1 (39.7 ± 4.0% vs. 0.9 ± 0.3%), CD200 (61.3 ± 3.1% vs. 34.6 ± 7.0%), HO-1 (93.5 ± 0.7% vs. 70.1 ± 12.8%), HLA-E (21.2 ± 2.1% vs. 1.0 ± 0.2%), and HLA-I (ABC) (97.4 ± 0.3% vs. 4.4 ± 0.9%). GStemHep cells cultured in inflammatory conditions vs. cells cultured in basal conditions significantly expressed higher levels of PD-L1 (39.7 ± 4.0% vs. 3.0 ± 0.6%), HLA-E (21.2 ± 2.1% vs. 0.9 ± 0.2%), CD200 (61.3 ± 3.1% vs. 54.2 ± 5.6%), HO-1 (93.5 ± 0.7% vs. 94.6 ± 0.9%), HLA-E (21.2 ± 2.1% vs. 0.9 ± 0.2%) and HLA-I (ABC) (97.4 ± 0.3% vs. 17.6 ± 2.6%). The absence of HLA-DR and HLA-G expression was maintained in both culture conditions vs. thawed cells, as was the high percentage of cells expressing the immunoregulatory molecules CD47 and IDO.

Analysis of the levels of expression for these molecules showed that culture of GStemHep in inflammatory conditions vs. thawed cells significantly increased the expression of CD47 and HLA-I (ABC). Cells cultured in inflammatory conditions vs. cells cultured in basal conditions showed greatly increased expression of HLA-I (ABC). The other markers, CD200, IDO, PD-L1, HLA-E, were not modified by culture in inflammatory conditions (**Supplementary Figure S1B**).

Thus, in an inflammatory context, the immunoregulatory profile of GStemHep was accentuated by the expression of new immunoregulatory molecules such as PD-L1, CD200, HO-1 and HLA-E, as well as in the expression levels of CD47, and maintained high expression of IDO. However, this was also accompanied by re-expression of HLA-I (ABC) molecules that had been repressed during differentiation.

### GStemHep evade T lymphocytes activation and proliferation *in vitro*

**Figure 3:**
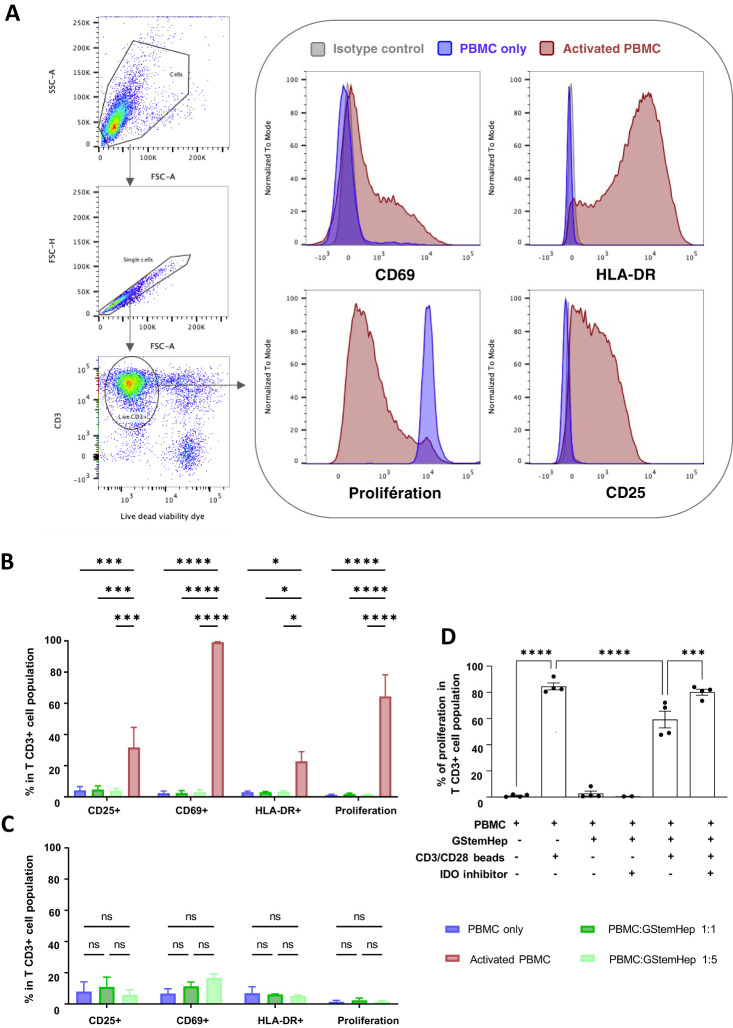
Activation and proliferation responses of T cells in contact with GStemHep *in vitro*. GStemHep were seeded and cocultured with PMBCs from fresh blood for 3 days. PBMC alone were used as a negative control. PBMC incubated with phorbol 12-myristate 13-acetate (PMA)/ionomycine or CD3/CD28 beads were used as a positive control. Activation (CD69, CD25 and HLA-DR) and proliferation (Cell Proliferation Dye) of gated CD3+ T lymphocytes was analyzed by flow cytometry. **(A)** Flow cytometry analysis strategy and representative histogram of negative (PBMC only, blue) and positive (activated PBMC, red) control. **(B)** Expression of activation and proliferation markers on CD3+ T cells at PBMC: GStemHep ratios 1:1 (n=6) and 1:5 (n=7) after coculture with GStemHep. The addition of PMA/ionomycin or CD3/CD28 beads is performed in the respective positive control (activated PBMC) of activation (n=4) and proliferation (n=6). **(C)** Expression of activation and proliferation markers on CD3+ T cells at PBMC: GStemHep ratios 1:1 and 1:5 after coculture with GStemHep in inflammatory condition (n=3) **(D)** Proliferation of CD3+ T cells in coculture with GStemHep (1:1 ratio) in the presence or absence of CD3/CD28 activation beads and IDO inhibitor (n=4). (two-way ANOVA test, P Value: *p < 0.05; ***p < 0.001; ****p < 0.0001; ns, not significant).

To assess T cell response against GStemHep, PBMCs and GStemHep coculture assays were performed. CPD-labelled human PBMCs were cultured for three days with GStemHep at several cell densities to assess the impact of cell ratio in culture medium with or without inflammatory cytokines. The flow cytometry analysis strategy and representative results of the experiments carried out are shown in **Figure 3A**. After gating of singlets and selection of live T cells (CD3+; Viability Dye-), activation of CD3+ T cells was followed by expression of the CD25, CD69 and HLA-DR markers, and proliferation by decrease in fluorescence intensity of the CPD marker.

Analysis of the T cell responses in coculture with GStemHep under non-inflammatory conditions is shown in **Figure 3B**. The activated PBMC condition used as a positive control for T cell activation markers was PMA/ionomycin stimulation and for proliferation CD3/CD28 bead stimulation. PMA/ionomycin activation of T cells resulted in significant expression of the activation markers CD69 (99.2 ± 0.2%), CD25 (31.7 ± 12.7%) and HLA-DR (22.8 ± 6.2%). Stimulation with CD3/CD28 beads resulted in 64.4 ± 13.9% proliferation of PBMCs. In coculture experiments, GStemHep did not activate T lymphocytes. Indeed, the percentage of T cells positive for the three activation markers studied is less than 5% overall, and equivalent to the negative control condition (PBMC alone) at 3 days (**Figure 3B**). The same negative results were observed when the response of T CD4+ and T CD8+ cells were analyzed (data not shown). Only 20% of T lymphocytes were CD69 positive after 5 days of coculture (data not shown). A 5-fold increase in the amount of GStemHep (ratio PBMC/GStemHep 1:5) had no effect on CD3+ T cell and activation and proliferation (1.4 ± 0.3%) vs. 1:1 ratio (1.9 ± 0.6%) and PBMC negative control (1.3 ± 0.4%) conditions.

Since HLA-I (ABC) is expressed by GStemHep under inflammatory conditions, potentially affecting the T cell responses, the coculture experiments were reproduced in this context (**Figure 3C**). As previously observed in basal conditions, no T cell proliferation and low expression of activation markers CD25 and HLA-DR equivalent to negative control (PBMC alone) were induced in inflammatory condition. A non-significant increase in CD69 expression at the 1:1 and 1:5 ratios was observed compared to control PBMC alone (12.1 ± 1.5% and 16.8 ± 4.4% vs. 6.8 ± 5.2%, respectively).

GStemHep express the IDO molecule, which has been shown to inhibit T cell proliferation by degrading available tryptophan. Its role in the T response against GStemHep was studied using an IDO inhibitor in PBMC/GStemHep coculture experiments with and without T activation by CD3/CD28 beads (**Figure 3D**).

As expected, culture with anti-CD3+/CD28+ beads resulted in T cell proliferation (84.5 ± 5.2%). T cell proliferation in response to anti-CD3+/CD28+ beads in the presence of GStemHep cells resulted in a significant decrease in proliferation (59.3 ± 12.8%, i.e. 25% inhibition). Inhibition of IDO restored T cell proliferation (80.1 ± 4.6%) to a level equivalent to that of T cells activated by CD23+/CD28+ beads without GStemHep. These results highlight the immunoregulatory activity of GStemHep on T cell proliferation, involving IDO.

Taken together, these results demonstrate a lack of proliferation and very low activation of CD3+ T lymphocytes in contact with GStemHep in basal or inflammatory conditions *in vitro*. GStemHep inhibited T cell proliferation under CD3+/CD28+ activation. This inhibition of T cell proliferation was partially mediated by the immunoregulatory molecule IDO.

### GStemHep induces macrophage phagocytosis *in vitro*

**Figure 4:**
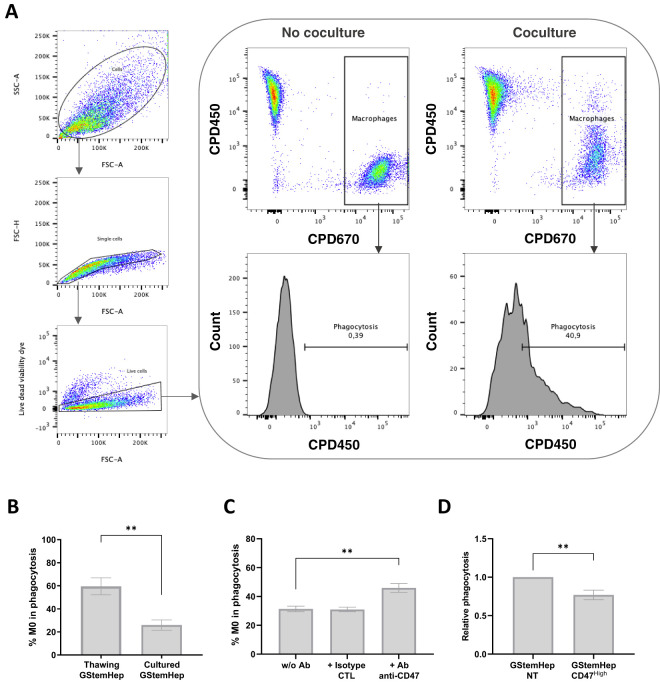
Phagocytosis response of macrophages in contact with GStemHep *in vitro*. Monocyte-derived macrophages labelled with CPD670 (CPD670+) were cultured for 2 hours with GStemHep CPD450+. Then, phagocytosis is rated by the percentage of M0 macrophages (CPD670+) that have phagocytosed GStemHep (CPD670+ CPD450+). **(A)** Flow cytometry analysis strategy and representative histogram of negative (no coculture) and positive (coculture) conditions. **(B)** Percentage phagocytosis of macrophages in contact with thawed (n=7) or freshly cultured (n=8) GStemHep (Mann-Whitney test, P Value: **p=0.0012). **(C)** Impact of CD47 blockade: Percentage of phagocytosis of macrophages in contact with GStemHep in antibody-free condition (w/o Ab), with isotype control antibody (CTL) or anti-CD47 antibody (n=8). **(D)** Impact of CD47 overexpression: Phagocytosis of M0 in coculture with GStemHep transduced with CD47 lentiviral vector (CD47^high^) relative to the condition with untreated GStemHep (NT) (n=11). (Wilcoxon test: **p<0.01).

To assess macrophage responses to GStemHep, phagocytosis assays were performed. Monocyte-derived macrophages and GStemHep were labelled with two different fluorochromes (CPD670 and CPD450, respectively) and cocultured for two hours (**Figure 4**). Phagocytosis was analyzed by flow cytometry, the analysis strategy and representative results in conditions with or without coculture are shown in **Figure 4A**. After gating of singlets and selection of live cells, phagocytosis was defined by the percentage of macrophages (CPD670+) that have phagocytosed GStemHep (CPD670+ CPD450+).

The percentage of phagocytosis was assessed directly on cryopreserved and thawed GStemHep cells or after thawing and 2-3 days of culture (**Figure 4B**). Phagocytosis of cultured cells averaged 25.9 ± 4.5%, increasing 2.3-fold with thawed GStemHep (59.6 ± 7.3%). It should be noted that thawed GStemHep cells showed ~20% of dead cells that could explain the higher phagocytosis as compared to cultured GStemHep cells that showed <5% dead cells.

A second microscopic technique was also performed to validate phagocytosis. Macrophages and GStemHep were labeled this time with fluorescent CellTracker and a fluorescent pH-sensitive pHrodo^®^ dye respectively. pH-sensitive dyes have a low fluorescence intensity at neutral pH, but upon acidification in the lysosome emits a several-fold higher fluorescence (32). After two hours of incubation, phagocytosis is observed by co-localization of fluorescence (**Supplementary Figure S2**).

Thus, GStemHep induced an innate immune response mediated by macrophages, despite their immunoregulatory profile and notably the expression of CD47, an inhibitor of phagocytosis by binding to SIRPα on the macrophage surface. The reduction of allograft rejection by CD47 overexpression in allogeneic PSCs and their derivatives has already been demonstrated (20, 33). Since we observed phagocytosis despite that around 80% of GStemHep cells expressed CD47, we analyzed the protective role on phagocytosis by macrophages of basal CD47 expression by GStemHep (**Figure 4C**), as well as after ectopic overexpression induced by cell transduction with a lentiviral vector expressing human CD47 (**Figure 4D**).

To assess the impact of basal CD47 in macrophage phagocytosis, an anti-CD47 blocking antibody was used in phagocytosis experiments to inhibit binding to SIRPα (**Figure 4C**). The GStemHep production batch used for these experiments (n=8) was CD47+ at 96%. The mean percentage of GStemHep phagocytosis was 31.4 ± 1%, and increased significantly to 45.9 ± 3.1% with the CD47-blocking antibody (no effect of control isotype) (**Figure 4C**). CD47/SIRPα binding is therefore responsible for a 15% decrease in phagocytosis in basal conditions. The CD47 molecule was then overexpressed in cultured GStemHep using a CD47-encoding lentiviral vector to assess whether the phagocytosis could be reduced. CD47 overexpression was validated by flow cytometry (**Supplementary Figure S3**). The GStemHep production used for these experiments is initially strongly positive for CD47 (94.4 ± 2.0%). After lentiviral transduction, 98.2 ± 1% of cells expressed CD47 with a 7 ± 0.4-fold increase in MFI (CD47^high^ cells) compared with non-transduced GStemHep (NT). The percentage of GStemHep NT and CD47^high^ phagocytosed by macrophages was then assessed (**Figure 4D**). Results are expressed as phagocytosis relative to the control (GStemHep NT), thus normalizing for inter-donor macrophage variability. The mean relative phagocytosis is 0.77 ± 0.06 with GStemHep CD47^high^ cells, thus, a decrease of 33% when the CD47 molecule is overexpressed.

To conclude, GStemHep induced an innate immune response mediated by macrophages and partially correlated with the expression of the CD47 on their surface. Overexpression of CD47 significantly reduced phagocytosis, but did not completely eliminate it.

### GStemHep induces NK cell activation and degranulation *in vitro*

**Figure 5:**
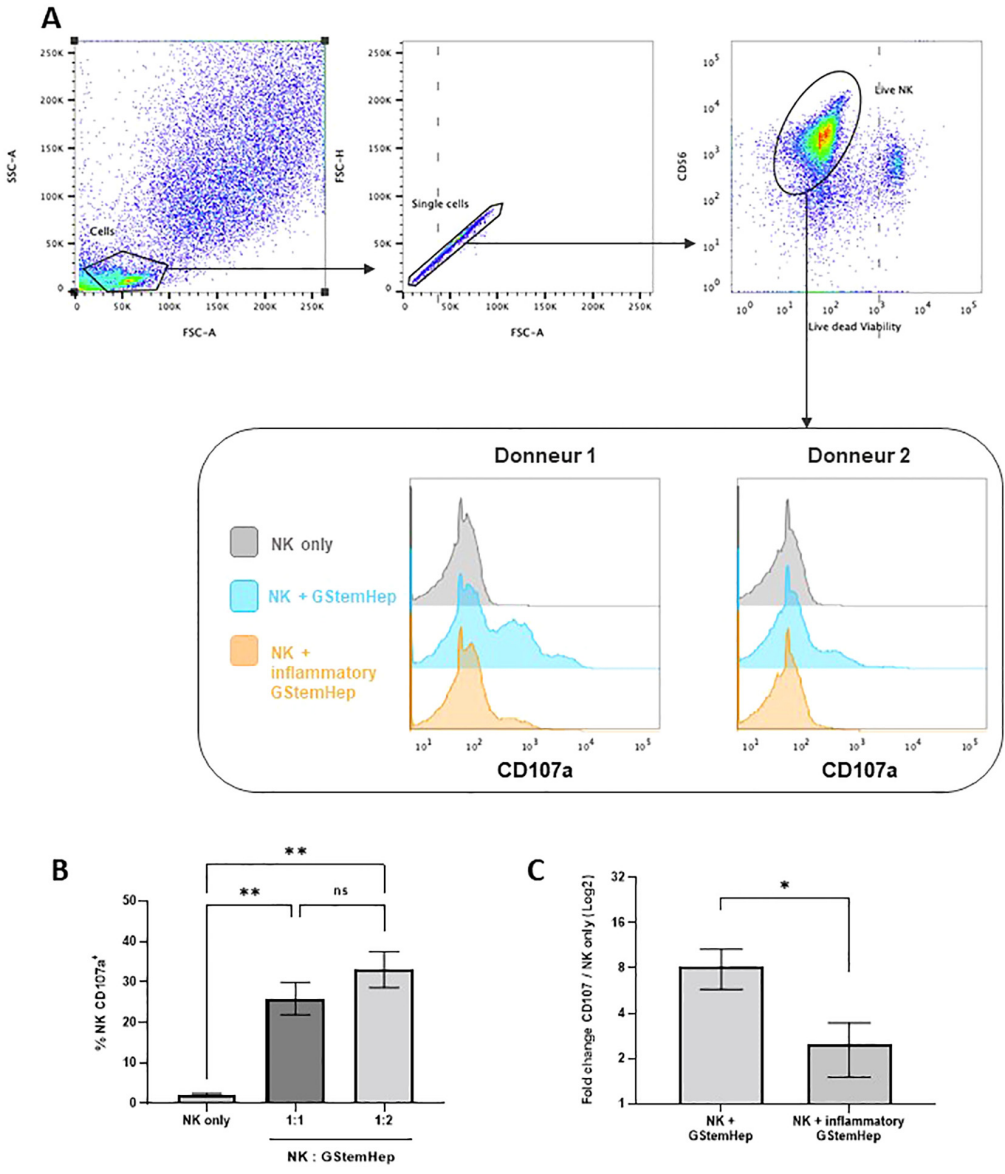
Cytotoxic response of NK cells in contact with GStemHep *in vitro*. Purified NK cells were cultured for 5 hours with GStemHep and an anti-CD107a antibody. Then, CD56+ NK cells were analyzed by flow cytometry for surface expression of the degranulation marker CD107a as an indicator of NK cell activation. Cultured NK cells alone were used as a negative control. **(A)** Flow cytometry analysis strategy and representative histogram of results on two donors for NK only, NK + GStemHep and NK + inflammatory GStemHep conditions. **(B)** Percentage of membrane CD107a expression on CD56+ NK cells in culture alone or cocultured with GStemHep at NK: GStemHep ratios 1:1 (n=8) and 1:2 (n=3) (Kruskal-Wallis test: **p < 0.01; ns: not significant). **(C)** Fold change in CD107a percentage of NKs in the presence of GStemHep (1:1 ratio) cultivated or not in inflammatory medium versus NK control alone (n=5) (Wilcoxon test: *p < 0.05).

Finally, we assessed the activation of NK cells towards GStemHep cells by performing analysis of CD107a as a marker of activation and degranulation of NK cells (**Figure 5**). Human NK cells purified from blood PBMCs were cocultured for five hours with GStemHep cells at two ratios of NK: GStemHep (1:1 and 1:2). After coculture, CD56+ NK cells were analyzed by flow cytometry for surface expression of CD107a. The flow cytometry analysis strategy and representative results are shown in **Figure 5A**. After gating of singlets and selection of live NK cells (CD56+; Viability dye -), activation is followed by CD107a expression. In the absence of GStemHep, the percentage of CD107a+ NK is very low or negative. Average CD107a+ expression and thus NK activation are shown in **Figure 5B**. These results were obtained from 8 experiments for the 1:1 ratio and 3 for the 1:2 ratio involving 7 different NK donors and 2 different GStemHep productions. Basal CD107a expression in NK cells cultured alone averaged 2.2 ± 0.2%. In co-culture with GStemHep, NK cell degranulation with CD107a membrane expression was significantly increased (25.9 ± 4.0% for the 1:1 ratio and 33 ± 4.4% for the 1:2 ratio) (**Figure 5B**). These results demonstrate an activation of NK cells against GStemHep cells, directly correlated with the amount of GStemHep cells.

NK cells target and eliminate cells lacking HLA class I (34). GStemHep cells after thawing express low levels (<5%) of HLA-I molecules, but that all cells express them under inflammatory culture conditions, with a fold change in MFI of 12.3 ± 1.7 (**Figure 2**, **Supplementary Figure S1B**). To assess the impact of HLA class I expression on the surface of GStemHep cells in inflammatory conditions in the NK responses, the degranulation assay was performed with GStemHep cells pre-cultured for 24h in medium with or without inflammatory medium (GStemHep vs Inflammatory GStemHep; n=4). To normalize for donor variability, CD107a expression in coculture conditions was plotted against the NK cells only condition (**Figure 5C**). The percentage of NK degranulation in the presence of GStemHep is 8.2 ± 2.4 fold greater than the condition without GStemHep. This ratio decreased significantly to 2.4 ± 0.8 in the presence of inflammatory GStemHep. These results demonstrate that the cytotoxic response of NK cells against GStemHep is primarily mediated by the absence of HLA class I on GStemHep.

## Discussion

Cell therapy with cell derivatives from allogeneic PSCs would result in CD8+ T cell and antibody-mediated rejection due to surface expression of HLA antigens (12, 15). Furthermore, cell transplantation through ischemia-reperfusion, necroptosis and the release of DAMPs can activate innate immune responses mediated by macrophages and NK cells (12, 15). The generation of hypoimmunogenic cells derived from allogeneic PSCs a likely requisite to avoid rejection. Several solutions have been proposed to this end, including inactivation of HLA-I (ABC) and HLA-II (35). HLA-I-deficient iPSCs and their derivatives is one of the most effective mechanisms to evade allogenicity but can trigger NK cytotoxicity via missing self-recognition mechanisms (36). The strategies to avoid this drawback have been the ectopic expression of tolerogenic non-classical HLA-I molecules, such as HLA-E (37) and HLA-G (38). These molecules are high-affinity ligands of the inhibitory CD94/NKG2A and LIR-1 receptors, expressed on NK cells but HLA-E can also bind to the NK activating CD94/NKG2C receptor (39). CD47 has also been shown to inhibit NK activation by HLA-I-deficient iPSC-derivatives (19, 20).

Differentiation from GStem cells to GStemHep cells was associated to an almost complete loss of HLA-I (ABC) expression, a phenomenon already observed in the differentiation from iPSC to pancreatic tissue (40). This is a favorable property with respect to avoiding T CD8+ cytotoxic and alloantibody immune responses. Nevertheless, this resulted in increased susceptibility to NK cell activation due to missing self-recognition mechanisms.

Inflammatory cytokines potentially released *in vivo* in pathological conditions and during transplantation resulted in re-expression of HLA-I molecules by GStemHep cells, particularly of HLA-I (ABC), but also in less proportion of HLA-E molecules, with a concomitant reduction of NK cell activation. Simultaneous avoidance of both T CD8+ and NK cell immune responses may need for T CD8+ cells to generate GStem cells deficient for HLA-I antigens, through deletion of beta-2 microglobulin (15). Inhibition of NK cells may need to express HLA-E (37) or HLA-G (38), ectopically increase CD47 expression (20), agonistics SIRPa engagers (41) or other checkpoint inhibitors (41, 42) as well as eliminate NK-activating ligands such as CD276 and CD155 (40). Alternatively, patients may receive an immunosuppressive treatment or more targeted biological treatments such as costimulation blockers or cell subset depleting antibodies (43).

Macrophages also play a role in the elimination of PSC derivatives and overexpression of the antiphagocytic CD47 by the transplanted cells reduced rejection (19). CD200 induces an immunosuppressive function through the binding to CD200R, which contains an inhibitory intracellular NPXY signaling motif (23). In models of transplantation, macrophage cytotoxicity and phagocytosis have been reduced through binding of CD200 in target cells to CD200R in macrophages (44). CD200 has even been described as even more potent than CD47 to inhibit macrophage activation (45). CD200 has also been described as an inhibitor of NK cells (24). Human macrophages also express inhibitory NK receptors, CD94/NKG2A and ILT-2,4 which recognize human HLA-E and HLA-G, respectively (46, 47) and could thus be ectopically expressed not only to inhibit NK cells but also macrophages. For GStemHep cells, we showed that they were phagocyted by macrophages and that spontaneous and ectopic increased CD47 expression by GStemHep cells partially protected them from phagocytosis. CD200 expression, albeit decreased during differentiation from GStem cells, was still high and further studies could define whether it also exerts a protective role against phagocytosis.

Absence of HO-1 expression by mouse iPSCs increases cell death by oxidative stress and accelerates differentiation (48). HO-1 expressed by stem cells of different origin and through production of heme catabolites derived of heme degradation has been shown to inhibit xenogeneic and allogeneic immune responses (29, 30). HO-1 has also been shown to promote hepatocyte survival (49, 50) and thus may have a dual beneficial effect on both hypoimmunogenicity and liver regeneration. GStemHep cells express high levels of HO-1 and further studies should define whether HO-1 and heme degradation products play a role important role in the protective action of GStemHep *in vivo* in ALF models.

IDO through catabolism of tryptophan has been clearly identified as an immunosuppressive mechanism on T cells and NK cells in different pathophysiological situations, including transplantation in general and also with stem cell-derivatives (26–28, 51). The expression of IDO by GStemHep cells and the experiments showing increased proliferation of T cells upon IDO inhibition indicate that GStemHep cells have an active immunoregulatory effect on T cells through IDO. IDO may be beneficial for immune evasion but it has been described as being a break for liver regeneration (52) and thus be detrimental for the *in vivo* effect on liver regeneration of GStemHep cells.

PD-L1 was not expressed neither by GStem nor GStemHep cells in basal conditions. This is on line with the absence of expression in PSCs (53) and human primary hepatocytes (54). PD-L1 expression was increased upon culture of GStemHep cells in inflammatory conditions and this may reinforce their immunoregulatory function *in vivo*.

Previously published results described that adult human liver stem cells were negative for HLA-G and HLA-II and express low levels of HLA-I (ABC), CD200 and HO-1. Upon differentiation to precursors of hepatocytes, maintained negativity HLA-G and HLA-II, as well as expression for HLA -I (ABC) and HO-1 and loose CD200 expression, while there was no description on their expression of IDO or CD47 (55–57). GStemHep (HLA-ABC^-^HLA-E^-^PD-L1^-^CD200^+^HO-1^+^IDO^+^CD47^++^) differ from adult human liver stem cells and primary human hepatocytes as they do not express HLA -I (ABC). This is likely either due to our hepatic differentiation protocol or the fact that HLA expression was only analyzed in previous studies at the hepatocyte-like cells stage, a stage more differentiated than that of GStemHep cells (56, 57).

Thus, GStemHep resemble the phenotype of hepatic stem cells but with very low or no repression of HLA-I (ABC). Upon inflammation, the phenotype of GStemHep cells was modified (HLA-ABC^+++^HLA-E^+^PD-L1^+^CD200^++^HO-1^++^IDO^++^CD47^+++^). In inflammatory conditions containing IFNγ and as previously described for PSC-derived hepatocytes (56), HLA-I antigens and PD-L1 significantly increased their expression, as well as for HLA-E albeit in lower percentages. Interestingly, HLA-II expression was not increased. Expression of the potent immunoregulatory molecules IDO and HO-1 was maintained at high levels.

The various immunomodulatory properties of GStemHep shown in the present study, are likely be part of their mode of actions in the treatment of acute liver failure, since we observed a rapid reduction of liver inflammation after injection of a single dose of GStemHep in mice intoxicated with acetaminophen (7). These immunomodulatory properties may also be useful, along with GStemHep’s cryoprotective and regenerative effects, for the treatment of chronic liver diseases by extending *in vivo* persistent of GStemHep in the context of allogenic cell transplantation. Multiple doses of GStemHep could be injected in patients with chronic liver cirrhosis or acute-on-chronic liver failure (ACLF) without anti-rejection immunosuppressive drugs, as it was performed in multiple doses with allogeneic adult-derived human liver stem cells and mesenchymal stem cells also expressing immunomodulatory molecule, without significant adverse effects (55, 58, 59). Despite these encouraging results allogenic cell rejection may be dependent of the cell dose, route of cell injection and patient’s liver stage disease.

## Limitations of the study

*In vivo* studies would give further insights into the immunogenicity of GStemHep cells. Various humanized immune system rodent models exist (60, 61) and could be used in the future to assess the immunogenicity of GStemHep transplanted cells and the acute and eventually chronic rejection mechanisms in an allogeneic situation.

Other rejection mechanisms of both acute and chronic graft rejection (12, 62) need to be considered in the future, mainly those dependent on antibodies, such as antibody-dependent cellular cytotoxicity, antibody-dependent cellular phagocytosis and antibody–mediated complement activation.

## Conclusions

GStemHep cells show an hypoimmunogenic phenotype and low activating levels of T cells, monocytes and NK cells. Inflammatory conditions reinforce the expression of some immunoregulatory molecules and although induced expression of HLA -I (ABC) and E expression, potentially inducing cytotoxic T cells, but GStemHep cells suppressed T cell proliferation *in vitro* and HLA -I (ABC) and E expression inhibited NK cell activation. These results support the use of GStemHep cell as off-the shelf allogeneic cells in patients with ALF and ACLF.

## Data Availability

The original contributions presented in the study are included in the article/**Supplementary Material**. Further inquiries can be directed to the corresponding author/s.
