# Supplementary material for: Human pluripotent stem cell-derived hepatic progenitors exhibit a partially hypoimmunogenic phenotype and actively inhibit immune responses
Source: Front Immunol. 2025 Feb 25;16:1507317. doi: 10.3389/fimmu.2025.1507317 (PMC11893836; doi:10.3389/fimmu.2025.1507317)
Supplement: Supplementary file 1 [file Presentation1.pptx]

## Slide 1
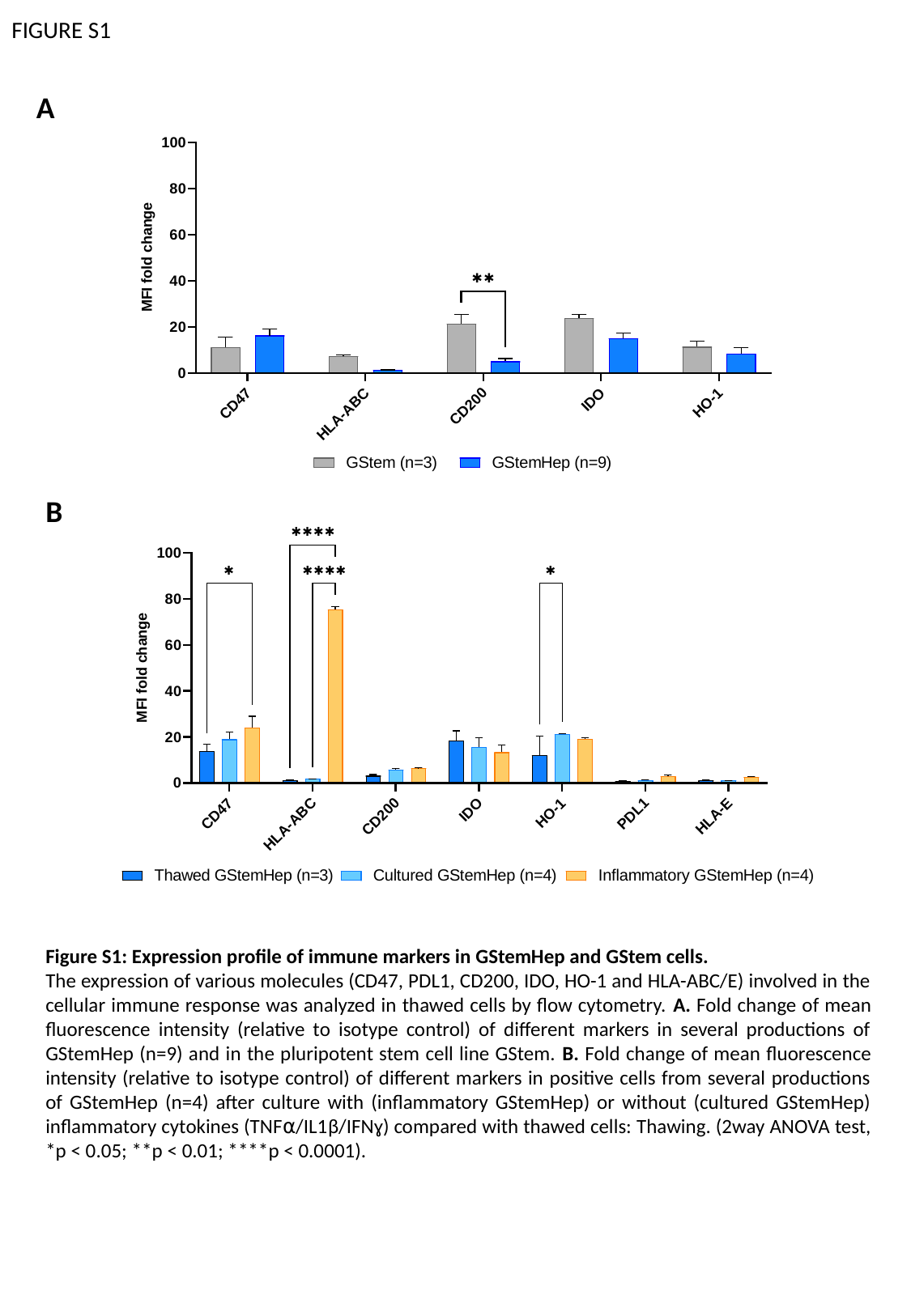

FIGURE S1
A
B
Figure S1: Expression profile of immune markers in GStemHep and GStem cells.
The expression of various molecules (CD47, PDL1, CD200, IDO, HO-1 and HLA-ABC/E) involved in the cellular immune response was analyzed in thawed cells by flow cytometry. A. Fold change of mean fluorescence intensity (relative to isotype control) of different markers in several productions of GStemHep (n=9) and in the pluripotent stem cell line GStem. B. Fold change of mean fluorescence intensity (relative to isotype control) of different markers in positive cells from several productions of GStemHep (n=4) after culture with (inflammatory GStemHep) or without (cultured GStemHep) inflammatory cytokines (TNF⍺/IL1β/IFNɣ) compared with thawed cells: Thawing. (2way ANOVA test, *p < 0.05; **p < 0.01; ****p < 0.0001).

## Slide 2
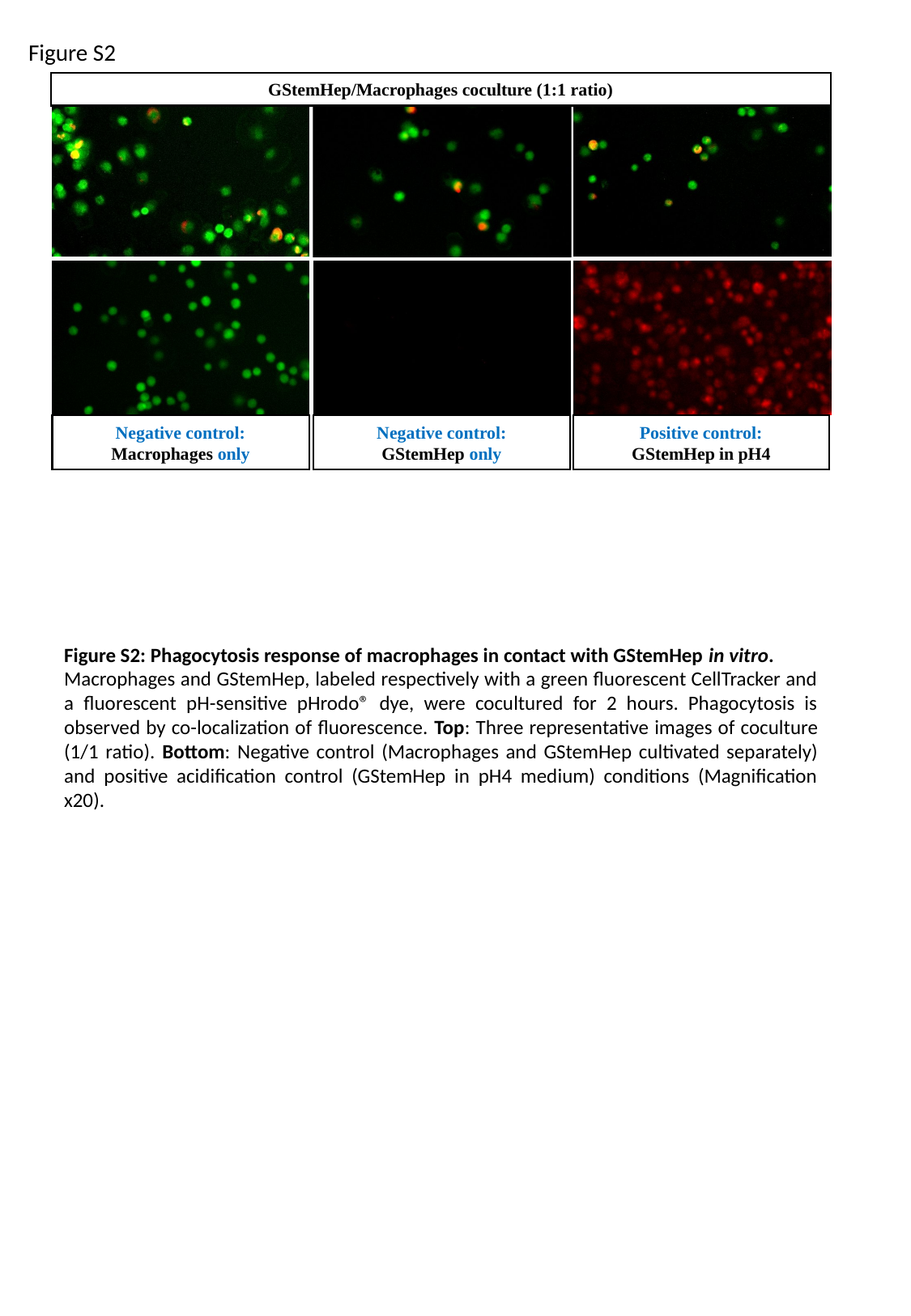

Figure S2
GStemHep/Macrophages coculture (1:1 ratio)
Negative control:
Macrophages only
Negative control:
GStemHep only
Positive control:
GStemHep in pH4
Figure S2: Phagocytosis response of macrophages in contact with GStemHep in vitro.
Macrophages and GStemHep, labeled respectively with a green fluorescent CellTracker and a fluorescent pH-sensitive pHrodo® dye, were cocultured for 2 hours. Phagocytosis is observed by co-localization of fluorescence. Top: Three representative images of coculture (1/1 ratio). Bottom: Negative control (Macrophages and GStemHep cultivated separately) and positive acidification control (GStemHep in pH4 medium) conditions (Magnification x20).

## Slide 3
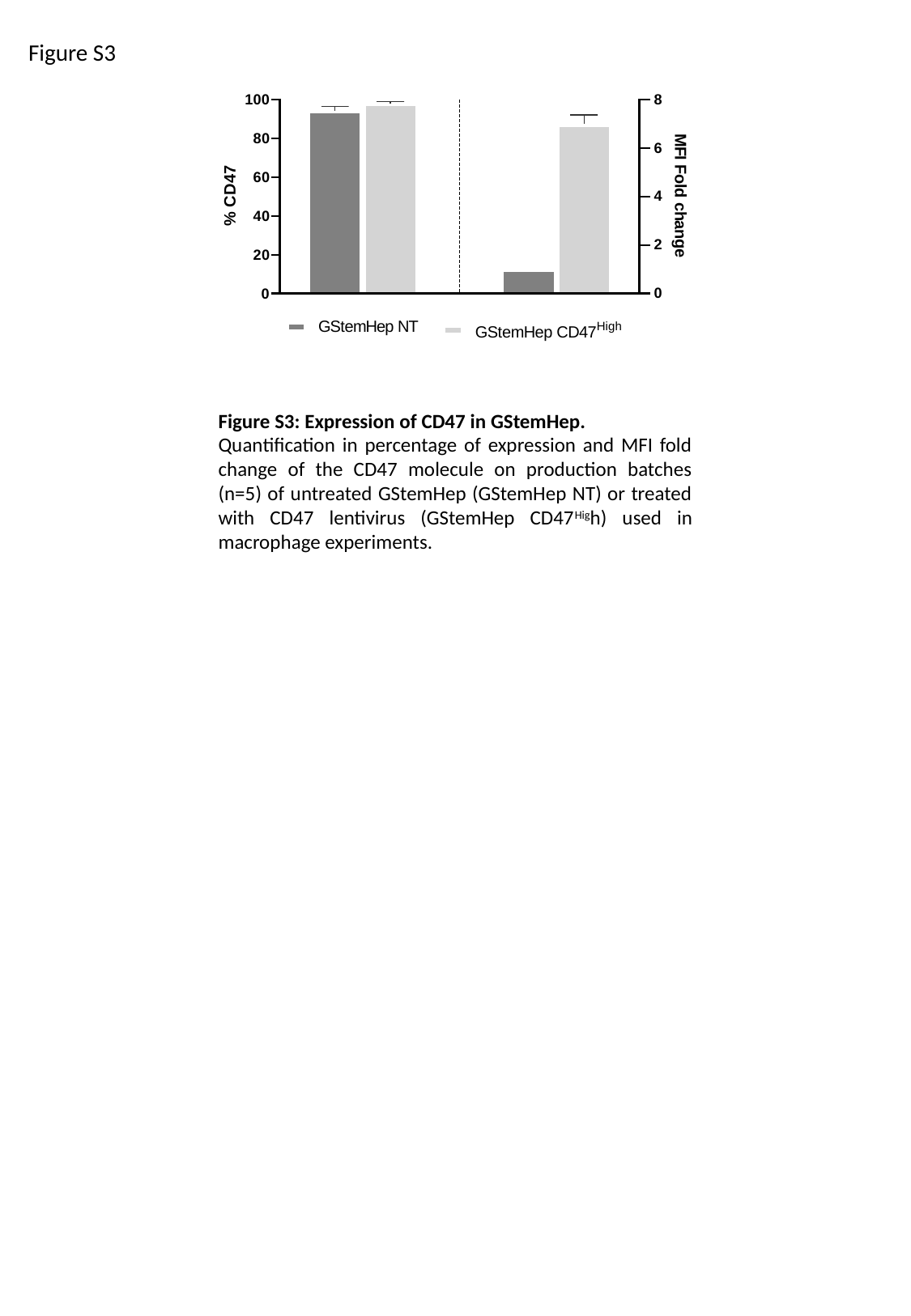

Figure S3
Figure S3: Expression of CD47 in GStemHep.
Quantification in percentage of expression and MFI fold change of the CD47 molecule on production batches (n=5) of untreated GStemHep (GStemHep NT) or treated with CD47 lentivirus (GStemHep CD47High) used in macrophage experiments.

## Slide 4
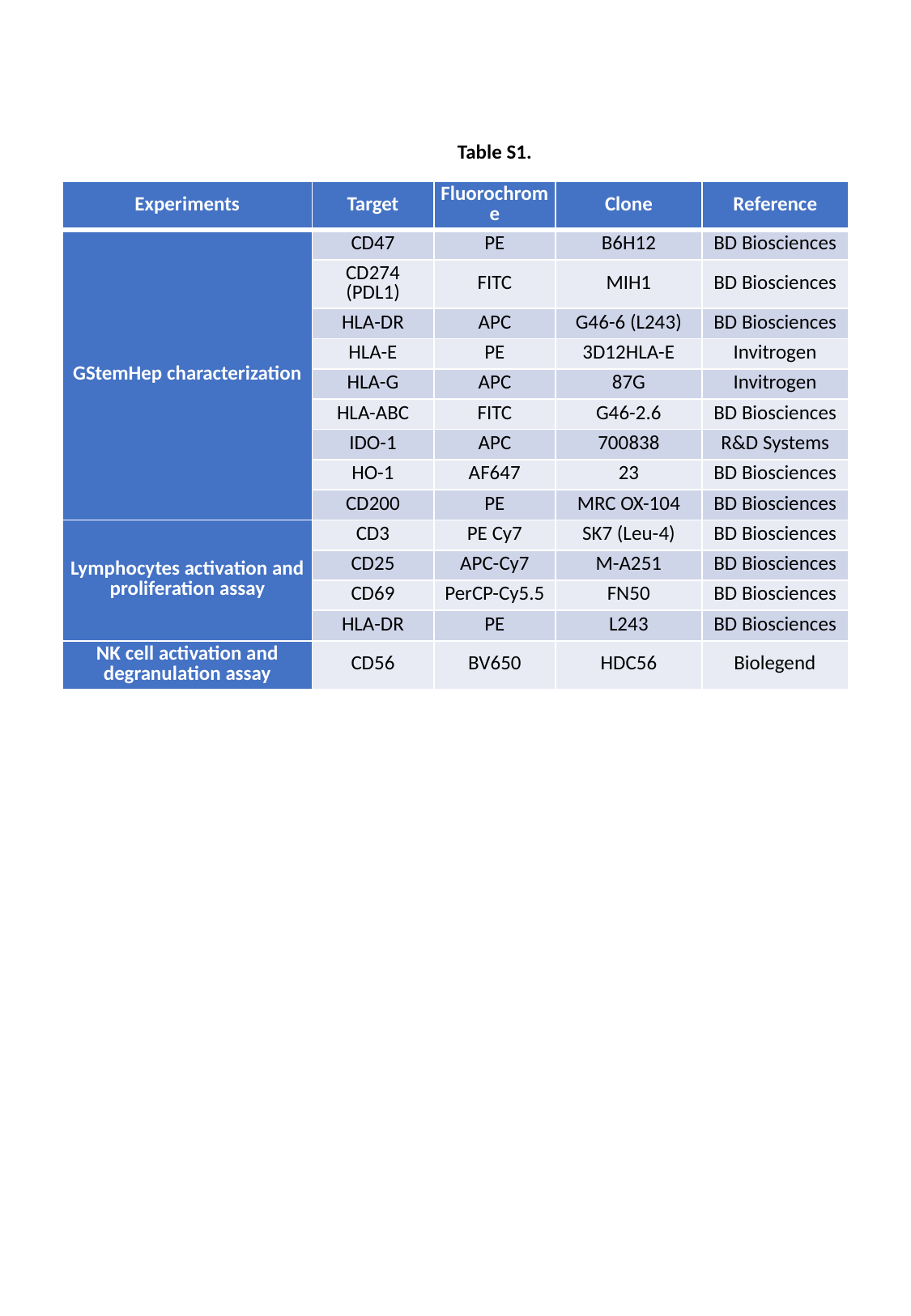

Table S1.
| Experiments | Target | Fluorochrome | Clone | Reference |
| --- | --- | --- | --- | --- |
| GStemHep characterization | CD47 | PE | B6H12 | BD Biosciences |
| | CD274 (PDL1) | FITC | MIH1 | BD Biosciences |
| | HLA-DR | APC | G46-6 (L243) | BD Biosciences |
| | HLA-E | PE | 3D12HLA-E | Invitrogen |
| | HLA-G | APC | 87G | Invitrogen |
| | HLA-ABC | FITC | G46-2.6 | BD Biosciences |
| | IDO-1 | APC | 700838 | R&D Systems |
| | HO-1 | AF647 | 23 | BD Biosciences |
| | CD200 | PE | MRC OX-104 | BD Biosciences |
| Lymphocytes activation and proliferation assay | CD3 | PE Cy7 | SK7 (Leu-4) | BD Biosciences |
| | CD25 | APC-Cy7 | M-A251 | BD Biosciences |
| | CD69 | PerCP-Cy5.5 | FN50 | BD Biosciences |
| | HLA-DR | PE | L243 | BD Biosciences |
| NK cell activation and degranulation assay | CD56 | BV650 | HDC56 | Biolegend |
